# Editorial: Multiomics approaches in the central nervous system and neuropathies

**DOI:** 10.3389/fnins.2025.1670706

**Published:** 2025-08-27

**Authors:** Emil Kriukov, Daisy Y. Shu, Wenjun Yan, Giulia Romano, Wai Lydia Tai

**Affiliations:** ^1^Schepens Eye Research Institute of Massachusetts Eye and Ear, Boston, MA, United States; ^2^Harvard Medical School Department of Ophthalmology, Boston, MA, United States; ^3^School of Optometry & Vision Science, University of New South Wales, Sydney, NSW, Australia; ^4^Department of Ophthalmology, School of Medicine, Stanford University, Stanford, CA, United States; ^5^International Centre for Genetic Engineering and Biotechnology, Trieste, Italy

**Keywords:** CNS, scRNAseq, multiomics, neuropathy, sequencing

The field of multiomics is constantly and rapidly evolving, with both well-established and emerging methodologies increasingly applied to solve complex biological problems on a computational basis. This systems-level approach spans multiple tissues and has been used to study the central nervous system (CNS) and associated neuropathies. This Research Topic of Frontiers in Neuroscience comprises three original research articles and one mini-review that collectively reflect the dynamic intersection of multiomics with neurobiology, from epigenetic regulation to seizure modeling and machine learning integration in brain immunology.

The work by Fang et al. investigated fragile X syndrome (FXS), a neurological disorder caused by epigenetic silencing of the FMR1 gene, using well-established-omics methods, such as bisulfite sequencing and ChIP assay, to help identify the factors that mediate silencing of the FMR1 gene. The authors focused on one of the nine identified candidate silencing factors, EZH2, and demonstrated that inhibition of EZH2 by a small molecule inhibitor corrects electrophysiological abnormalities in cultured fragile X syndrome neurons. Further, *in vivo* administration of antisense oligonucleotide targeting *EZH2* reactivates FMR1 expression in human FXS neural progenitor cells engrafted within the brains of mice. This study showcased a sophisticated approach of the “dry-to-wet biology” funnel, where sequencing of the epigenome helped to identify potential target genes that were verified *in vitro* and *in vivo*.

Popova et al. demonstrate the application of mRNA- and microRNA-seq to analyze temporal changes in the mouse hippocampus transcriptome after pilocarpine-induced seizures. The authors characterize the longitudinal molecular changes in the mouse hippocampus at 1, 8, 36, and 120 hours upon the induction of status epilepticus. The analysis focused on differentially expressed genes and pathways enriched in the studied condition, describing major temporal changes in the transcriptome, which was supported by microRNA changes shown in microRNA-seq. Overall, this study demonstrates a comprehensive effort to systematically address knowledge gaps in a well-established disease model condition.

Such comprehensive efforts were also performed by Sha et al. on bulk and single-cell RNA sequencing (scRNA-seq) levels in Alzheimer's disease (AD) to study potential regulation of cellular senescence. While the study itself does not generate new raw sequencing data, it is a great demonstration of leveraging publicly available sequencing datasets for discovery. The authors describe cellular senescence by exploring the activity score of related genes using AUCell. This, being one of the approaches to drive senescence parameters from scRNA-seq data, showed that astrocytes, microglia, and vascular cells are the most active populations. The authors further constructed a non-coding RNA regulatory network to explore how the non-coding RNAs (e.g., microRNA and long non-coding RNA) can regulate AD-associated senescence genes.

To summarize recent developments in the -omics field and its application in brain immunology, Binder et al. review advances in single-cell data analysis approaches, particularly regarding autoencoders and graph neural networks. The authors refer to several classical packages such as Seurat and scanpy, scVI VAE, and describe the recent advances in foundation models. The last is a growing trend, focused on “atlas” generation, that serves as a tool in many ways: whether it is a reference atlas for automated data annotation and mapping, or the object to analyze itself. The authors discuss the current shift in the methodology from canonical CPU-based algorithms to machine learning approaches. While this seems to be a promising large-scale high-throughput direction, proper connection between computationally-derived results and biological reality remains essential.

Together, these four contributions highlight the power of multiomics to unravel complex disease mechanisms and shape new directions for neuroscience research. Whether through data integration across temporal disease modeling or atlas generation, the field continues to push the boundaries of how we understand the brain in health and disease. We hope that this Research Topic inspires further cross-disciplinary collaborations and drives new research initiatives at the interface of neuroscience and multiomics technologies.

